# Cluster of liver cancer and immigration: A geographic analysis of incidence data for Ontario 1998–2002

**DOI:** 10.1186/1476-072X-7-28

**Published:** 2008-06-02

**Authors:** Yue Chen, Qilong Yi, Yang Mao

**Affiliations:** 1 Department of Epidemiology and Community Medicine, Faculty of Medicine, University of Ottawa, 451 Smyth Road, Ottawa, Ontario, Canada; 2 Centre for Health Surveillance, Chronic Disease Prevention and Control, Public Health Agency of Canada, Ottawa, Ontario, Canada

## Abstract

**Background:**

Liver cancer is not common in Canada in general; however, clustering of the disease causes a concern. We conducted a spatial analysis to determine the geographic variation of liver cancer and its association with the proportion of immigration in Ontario. Liver cancer incidence data between 1998 and 2002 were obtained from the Ontario Cancer Registry. The Canadian Community Health Survey (CCHS) in 2001 provided information on potential risk factors.

**Results:**

Age standardized incidence ratios (SIR) for liver cancer and prevalence of potential risk factors were calculated for each of 35 health regions. The SIRs for liver cancer varied across the 35 health regions (p < 0.01). Toronto and York health regions had a significantly higher SIR than other regions, indicated by the Scan method (p < 0.001). Poisson models with and without random effects were fitted to determine independent ecological contributors. After adjustment for sex, age and spatial location, the proportion of immigrants remained a significant determinant. Smoking, alcohol drinking, physical activity, education, income, obesity and diabetes did not substantially explain the geographic variation of liver cancer in Ontario.

**Conclusion:**

Immigration is an important reason for the clustering of liver cancer in Ontario. More attention should be paid to areas with a high proportion of immigrants.

## Background

Liver cancer is the sixth most common cancer worldwide and the third most common cause of death from cancer[1]. Liver cancer incidence has been increasing in developed countries [2-6] including Canada[7], but 82% of cases (and deaths) are in developing countries[1]. Hepatitis B and C viruses are major risk factors for liver cancer, and more than 75% of cases worldwide, and 85% of cases in developing countries, are caused by these two viruses[1,8,9]. Liver cancer affects Asian/Pacific Islander Americans disproportionately in the U.S.[10,11]. In Canada, the age-standardized mortality rate for liver cancer is most significantly elevated for birthplace of Asia and to a lesser degree for Europe[12]. However, a study from Houston, Texas, suggests that the increase in liver cancer risk is not from immigration but represents a true rise among the native born population[13].

Ontario, located in the centre part of Canada, has a population of 12 million or 38% of the total Canadian population. The sex and age standardized incidence rate (/100,000) of liver cancer was 3.18 in 2004 compared to 2.18 in. In this analysis, we used the data collected by the Ontario Cancer Registry in Ontario for the period from 1998 to 2002 and the 2001 Canadian Community Health Survey (CCHS). We examined the geographic distribution of liver cancer incidence and assessed the impacts of area level measures of immigrant, smoking, alcohol use, obesity, and diabetes in Ontario, Canada.

## Results

For the study period from 1998 to 2002, there were a total of 1877 liver cancer cases among Ontarians 15 years of age or more, based on data from the Ontario Cancer Registry. The average age of cases was 65.1 ± 7.4 years. Approximately 30.6% of cases were under 60 years of age; 73.8% were men. Table 1 shows the mean and range of liver cancer incidence and population characteristics across the health regions in Ontario in 2000–01.

**Table 1:** Average and range of liver cancer incidence and population characteristics of 35 health regions in Ontario, Canada, 2000–2001

	Average	Minimum – maximum	Coefficient of variance
Incidence of liver cancer (/100,000)	3.99	1.1–6.3	0.34
Immigrant (%)	30.5	3.1–55.9	0.72
Male (%)	48.9	48.1–50.1	0.01
Current smoking (%)	25.5	22.2–33.5	0.11
Alcohol drinking (%)	78.0	71.6–85.9	0.04
Physically active (%)	21.8	16.5–32.4	0.17
Obesity (%)	14.5	0.7–21.8	0.16
Reduced fruit and vegetable intake (%)	62.2	55.3–70.8	0.06
Diabetes (%)	4.4	3.5–6.5	0.19
High education (%)	46.4	35.5–57.5	0.11
Low income (%)	9.5	3.9–18.5	0.30

**Figure 1:**
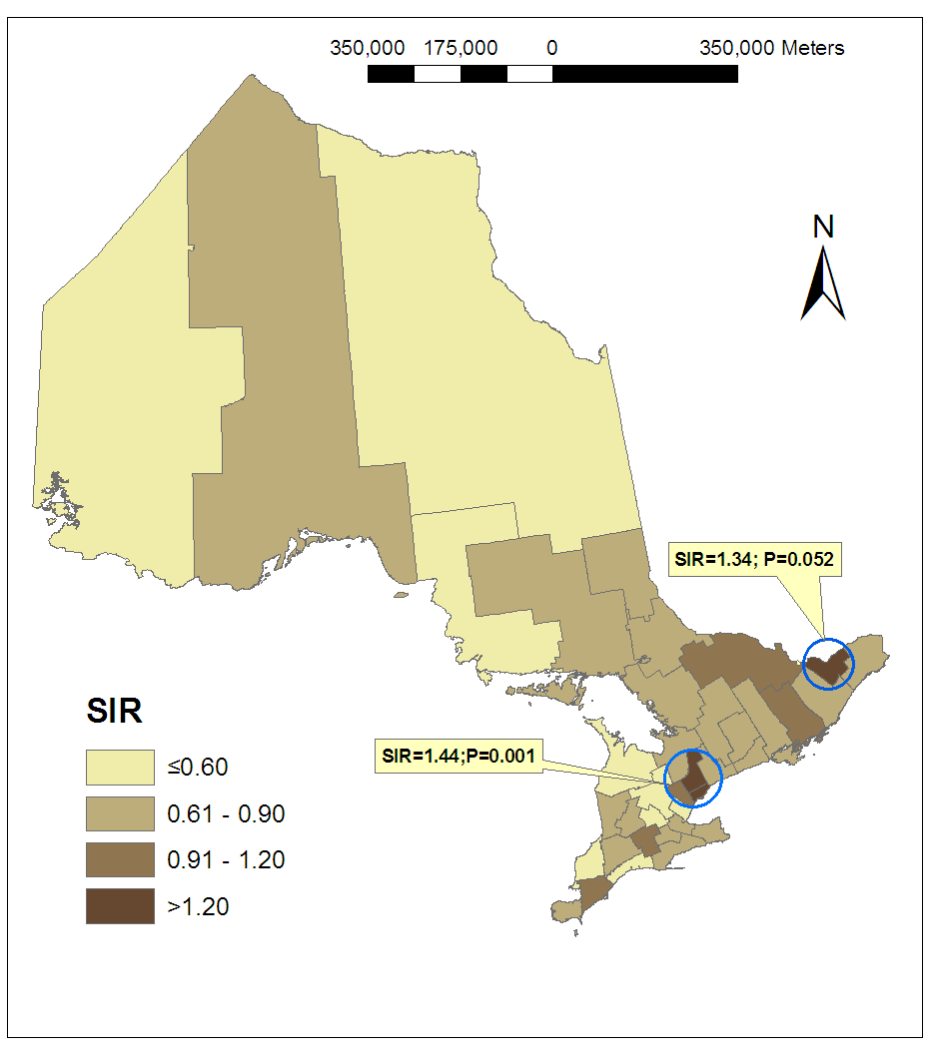
Regional distribution of standardized incidence ratio of liver cancer for Ontarians aged 15+ years: 1998–2002.

Figure 1 shows the geographic distribution of the age and sex standardized incidence ratios (SIRs) by health regions in Ontario. Using the scan method, we identified two clusters of high incidence for liver cancer in Ontario: one cluster included three health regions (Toronto, York and Peel) with a SIR of 1.44 (p = 0.001) and another one included Ottawa health region (p = 0.052).

Table 2 shows the results of spatial correlation for liver cancer from hierarchical Poisson models. Moran's I and Geary's C described the spatial correlation among the neighbouring regions, and show consistent results. When only age and sex were included in the analysis (Model 1), both indexes were significant. After adjustment for geolocation, both Moran's I and Geary's C were closer to null values but remained significant (Model 2). After further adjustment for the proportion of immigrants (Model 3), neither Moran's I nor Geary's C was significant, indicating that the spatial correlation among neighbouring regions could be explained by the proportion of immigration. Regional random effects reflect the variation among the health regions. The variance was significant with or without adjustment for the selected covariables (Models 1–4).

**Table 2:** Overall spatial correlation for liver cancer across 35 health regions in Ontario – Hierarchical Poisson models

	Variables Adjusted	Variance (SE) for RRE^a^	Moran's I^b ^(p value)	Geary's C^b ^(p value)
Model 1	Age, sex	0.086 (0.028)	0.231 (0.006)	0.611 (0.002)
Model 2	+ geolocation	0.080 (0.027)	0.182 (0.020)	0.677 (0.010)
Model 3	+ immigration	0.049 (0.021)	0.062 (0.198)	0.818 (0.094)
Model 4	+ selected factors^c^	0.050 (0.022)	0.073 (0.172)	0.810 (0.085)

^a ^Variance and standard error (SE) for regional random effects (RRE), all significant at the level p < 0.01;

^b ^Calculated from estimated regional random effects.

^c ^Including smoking, alcohol drinking, exercise, obesity, consumption of fruits and vegetables, diabetes, education and income.

**Figure 2:**
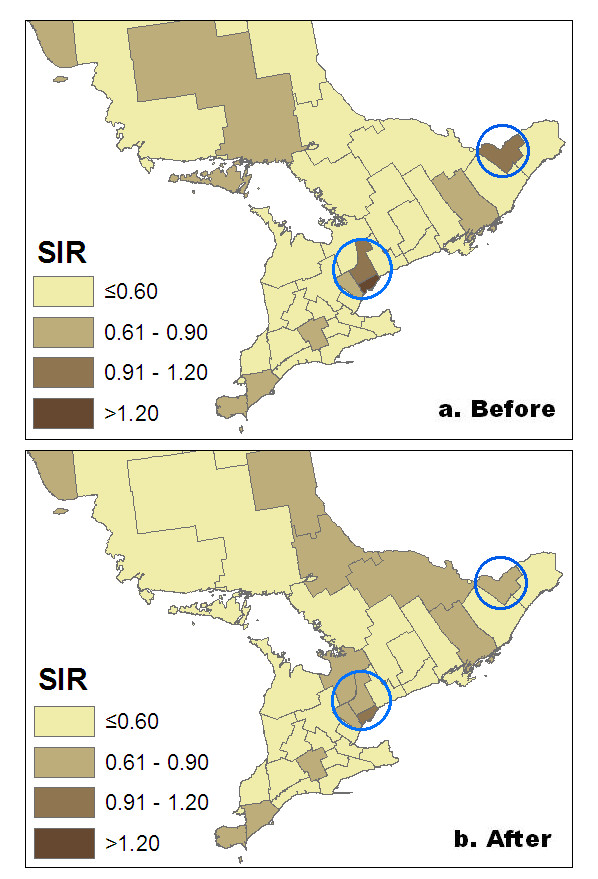
Relative risk for liver cancer across 35 health regions in Ontario: Before and after adjustment for the proportion of immigrants in addition to age, sex, and geolocation.

Table 3 presents the results from Model 3. The proportion of immigrants and longitude were significantly associated with the incidence of liver cancer at the health region level after adjustment for sex, age and their interaction. The proportion of immigration was notably high in the Toronto metropolitan area (data available upon request). Figure 2 shows the relative risk for liver cancer across the 35 health regions in Ontario before and after the proportion of immigration has been taken into consideration in addition to age, sex and geolocation. There were no notable changes when we accounted for other risk factors in the Poisson model (Model 4) and spatial correlation in the CAR model (data available upon request).

**Table 3:** Regression coefficients (β) and standard errors (SE_β_) for liver cancer in Ontario – Poisson regression analysis

Variables	β	SE_β_	p value
Intercept	-4.9339	1.6249	0.005
Age (years)			
15–49	-1.991	0.320	<0.001
50–59	0.334	0.303	0.270
60–69	0.645	0.277	0.021
70–79	0.913	0.272	<0.001
80+	(Reference)		
Sex			
Male	(Reference)		
Female	-0.745	0.1598	<0.001
Sex × age interaction			
15–49	-0.463	0.220	0.037
50–59	-0.949	0.219	<0.001
60–69	-0.470	0.189	0.013
70–79	-0.343	0.182	0.061
Proportion of immigrants	0.00791	0.00248	0.002
Geolocation			
Latitude	0.0106	0.0273	0.699
Longitude	0.0505	0.0208	0.015

## Discussion

Our analysis demonstrated that the incidence of liver cancer in Ontario was not randomly distributed geographically. There was a significant variation among health regions and one high risk cluster included Toronto, York and Peel health regions. This geographic variation in liver cancer incidence did not result from differences in age and sex. Although elderly people and men had increased risk of liver cancer, the observed geographic variation remained when results were adjusted for age and sex.

We found that the proportion of immigrants was a main determinant for the geographic variation of liver cancer in Ontario. After the proportion of immigrants was taken into consideration, both Moran's I and Geary's C were no longer significant. Rosenblatt et al.[14] compared the incidence of primary liver cancer in Chinese, Japanese, and Filipino migrants to the U.S. with that of their descendants and U.S. born Whites. The incidence was higher for men born in Asia than Asian men born in the U.S., and U.S. Whites had the lowest rate. Luo et al.[15] examined the incidence of cancer among Chinese immigrants in Alberta, and found that the overall cancer incidence was lower among immigrants, but the incidence of liver cancer was much higher compared to Canadian-born Alberta residents. Globally, sub-Saharan Africa, eastern and southeastern Asia, and Melanesia have high rates of liver cancer[1].

Although it is known that hepatitis B and C viruses are major risk factors for liver cancer[1,8,9], the cause of rising incidence of liver cancer in both the U.S. and Canada remains unclear. Hepatitis C virus (HCV) infection can explain a substantial proportion of liver cancer incidence in the U.S. in recent years[16,17] and might also explain the differences between Danish and U.S. trends in liver cancer incidence[18]. In addition to drug use and blood transfusions[18], immigration can also be an major source of HCV. Recent immigrants from hepatitis B virus (HBV) endemic areas and their descendants are at high risk of chronic HBV infection, and HBV-related liver cancer[19], and it is expected to be similar for HCV.

Canadian census data show an in increase in immigration from Africa and Asia in recent decades. Immigrants from Africa and Asia made up about 17% of the foreign-born population in 1981, increasing to 28% in 1996 and 42% in 2001. Concurrently, immigrants from Europe made up a decreasing proportion of the foreign-born population, beginning at 67% in 1981 and dropping to 55% in 1996 and then 42% in 2001. There is a possibility that the increased incidence rate of liver cancer is related to the increased proportion of immigrants from Asia and Africa during the past several decades.

It has been documented previously that increased incidence of liver cancer is associated with smoking[20,21], heavy alcohol drinking[22,23] and obesity[24] In this analysis, we did not find that smoking, alcohol drinking, obesity, diabetes, fruit and vegetable consumption, physical activity, income and education at the health region level explained a significant part of the geographic variation of liver cancer incidence in Ontario.

The study has potential limitations. Within health regions, there are clusters for people with different ethnic backgrounds. In the city of Toronto for example, there is an increased proportion of immigrants from Asia who live in the Scarborough area. Smaller area units may result in finer results. This is related to the scale effects of the modifiable areal unit problem[25,26]. However, in this study there were no smaller regional data available. Although the association between immigration and liver cancer is important, there is no conclusion of causation from this study. We did not have information on important risk factors such as HBV or HCV infection. Other potential risk factors data were collected in 2001 while the cancer incidence was for the period from 1998 to 2002. We can only assume that the current exposure correlates the previous exposure, which may or may not be true. Although immigration status did not change over time, the proportion of immigrants in health regions might change. In addition, information on race and ethnicity is not available in the Canadian Cancer Registry data, and therefore, we do not know if these immigrants are more likely to come from areas where HBV and/or HCV are endemic. In this analysis, immigrant proportion explained most of the variation of region random effects, which decreased from 0.080 to 0.049. However, the residual variation remained significant even after other covariates were included in the model, indicating that there are some unidentified important factors for the regional difference. Heavy alcohol drinking is an important risk factor for liver cancer; however, the quantity and frequency of alcohol consumption could not be easily adjusted for at the ecological level.

## Conclusion

The significant geographic variations in the incidence of liver cancer in Ontario could largely be explained by differences in the proportion of immigrants. The prevalence of smoking, alcohol use, obesity and diabetes as well as physical activity, fruit and vegetable consumption, education and income were not significant contributors to the geographic variation. Further studies are needed to examine the reasons for the association between immigration and liver cancer, such as the prevalence of HBV or HCV infections among immigrants and non-immigrants. The study provides useful information that may help planning and distribution of resources. The results may also underscore the importance of efforts to promote screening and treatment programs for liver cancer in the regions with a high risk.

## Methods

### Data sources

Cancer incidence data were obtained from the Ontario Cancer Registry (OCR). The OCR has accumulated data for liver cancer since the 1960s. In this analysis we used data collected during the period from 1998 to 2002. Cancers were coded according to the International Classification of Diseases Second Edition (ICDO-2). Eligible cases for inclusion in this analysis were all individuals from 35 health regions in the province with primary liver cancer (ICDO-3 C22.0 excluding 9590:9989). We obtained number of cases and population for each region by gender and age groups from the OCR. We excluded cases under age of 15 years. Hepatocellular carcinoma is the most common type of primary liver cancer in Canada, accounting for about 85% of all cases. Prevalence of potential cancer risk factors were calculated based on data from the CCHS conducted from September 2000 to November 2001 (cycle 1.1). The target population of the survey was household residents aged 12 years or older in all the ten provinces and three territories in Canada, excluding individuals living on Indian Reserves or Crown lands, clientele of institutions, full-time members of the Canadian Armed Forces and residents of certain remote regions. The survey targeted approximately 98% of Canadians aged 12 years or more and included more than 130,000 subjects. The questionnaire was administered using computer-assisted interviewing. Sampling units selected from a telephone list frame were interviewed from centralized centres, and those selected from an area frame were mainly interviewed by decentralized field interviewers. In all selected dwellings, a knowledgeable household member was asked to supply basic demographic information on all residents of the dwelling. One member of the household was then selected for a more in-depth interview. The survey included questions related to health status, health care utilization and health determinants. To match with the cancer data, only data from people aged 15+ years living in Ontario were used in the analysis. In the public use micro data file, some regions were combined with a total of 35 health regions in Ontario. Based on the health region map, an adjacency matrix was created manually for 35 collapsed regions to reflect the neighborhood structure and used in the spatial analysis. Geographic centroids for each health region were calculated based on the map polygon.

The CCHS covered information on potential cancer risk factors. For our study, we obtained the proportion of individuals in each health region in Ontario with daily smoking, regular alcohol use, leisure time physical activity, obesity and diabetes. Current smokers were respondents who had smoked at least 100 cigarettes during their lifetime and reported smoking cigarettes every day or almost every day at the time of the survey. A regular alcohol drinker was defined as a person who drank alcoholic beverages at least once a month during the past twelve month. Active subjects were those that had total daily energy expenditure values of 3+ kcal/kg/day for leisure time physical activity. Obesity was defined as body mass index equal to or larger than 30 kg/m^2^. People were considered to have diabetes if they answered the following question with an affirmative response: "Do you have diabetes diagnosed by a health professional?" Other variables included in the analysis were income, education and stress.

### Data analysis

The standardized incidence ratio (SIR) for live cancer was calculated for each of the 35 health regions and mapped to visually show the geographic distribution. To detect the specific locations of either high rate or low rate clusters with a minimum of assumptions about cluster size, and to evaluate their statistical significance, we employed the spatial scan statistic proposed by Kulldorff[27,28]. This method imposes an infinite number of circles on the map, with circle centroids at any of the region coordinates and with different radius. We tested the difference in cancer incidence between inside and outside the circle. Each circle is a potential cluster that is least likely to have occurred by chance. Moran's I and Geary's C were calculated for the distribution of incidence ratio of liver cancer to assess the overall spatial correlation between neighbouring regions[29,30] Moran's I ranges from -1 to 1, with a positive/negative signage representing positive/negative spatial autocorrelation and zero representing no spatial autocorrelation. Geary's C ranges from 0 to +2, with zero being a strong positive spatial autocorrelation, through to 2 which represents a strong negative spatial autocorrelation.

For both Moran's I (or Geary's C) and the scan method describe above, p-values were obtained by using Monte Carlo hypothesis testing with an adjustment for multiple testing. Hierarchical Poisson models[31] were fitted to explore the association between aggregated factors and liver cancer incidence with health region treated as a clustering factor. The aggregated factors included alcohol use, smoking, fruits and vegetable consumption, obesity, immigrant status, income and education as well as geographic location of the health region (longitude/latitude was used as a proxy for the location of health regions). Both random effects (health regions) and fixed effects (alcohol use, smoking, exercise, diabetes, obesity, fruit and vegetable consumption, education and income) were analyzed simultaneously. A conditional autocorrelation model (CAR) was constructed to examine the impacts of those factors on liver cancer with both spatial correlation and regional random effects being considered.[32] All the analyses were conducted by using SAS (SAS Institute Inc., Cary, NC), SaTScan package  and WINBUGS software . Maps were created using ArcGIS package .

## Abbreviations

CAR model: conditional autocorrelation model; CCHS: Canadian Community Health Survey; HBV: Hepatitis B virus; HCV: hepatitis C virus; ICDO-2: International Classification of Diseases Second Edition; LTPA: leisure time physical activity; OCR: Ontario Cancer Registry; SIR: standardized incidence ratio; EE: Energy expenditure

## Competing interests

The authors declare that they have no competing interests.

## Authors' contributions

YC, QY, and YM contributed to the conception and design of the study, QY performed the statistical analysis, YC prepared the first draft and all authors contributed to the writing of the manuscript.
